# Field Enhancement for the Composite MXene/Black Phosphorus-Based Metasurface

**DOI:** 10.3390/nano12183155

**Published:** 2022-09-11

**Authors:** Yihui Zhou, Lingqiao Li, Zhihui He, Yixuan Wang, Wei Cui, Zhimin Yang, Shaojun Lu, Xiongxiong Wu, Lang Bai

**Affiliations:** 1 School of Physics and Electronic Information, Innovation Team of Smart Metamaterials for Weak Signal Detection, Yan’an University, Yan’an 716000, China; 2 School of Chemistry and Chemical Engineering, Yan’an University, Yan’an 716000, China

**Keywords:** black phosphorus, MXene, metasurface, plasmonics

## Abstract

Both MXene and black phosphorus (BP), which actg as hot two-dimensional (2D) materials, have unique optical properties and important applications for nano-micro optical devices. Here, a composite MXene/BP-based metasurface, consisting of Ti_3_C_2_T_x_ and BP layers, is proposed for investigating the optical responses and electric field by using the finite-difference time-domain numerical simulation method in the microwave band. The research results show that the Fano resonance-like spectra can be observed when the coupling of surface plasmons (SPs) on the BP and MXene layers appears. Furthermore, the field enhancement, based on the Fano resonance-like optical responses, can be improved by an order of magnitude through adjusting the structural parameters and the polarization direction of incident light for the proposed metasurface. The findings may provide important theoretical insights into the design and realization of high-performance plasmonic devices.

## 1. Introduction

Since the 21st century, a single atomic layer of graphene has been successfully isolated by Geim’s group; 2D materials have gradually become a research hotspot due to their advantages of nano-scale thickness, strong field confinement, easy controllability, and low loss [1,2,3]. Interestingly, SPs on the surface of many 2D materials have also been found in recent years [4,5,6]. Due to the symmetry of the lattice, SPs exhibit distinct propagation characteristics for different kinds of 2D materials [7,8,9]. The one type is isotropic 2D materials [10,11], such as graphene. SPs can be effectively excited on the graphene in the terahertz frequency range, and many interesting phenomenon can also be realized for the graphene optical system, such as plasmonic-induced transparency and Fano resonance [12,13,14,15]. The other type is anisotropic materials, such as BP [8,15,16]. BP acts as one of the most popular 2D materials and has been widely investigated owing to its advantage of in-plane anisotropy, SP excitation, and strong, confined electric field [17,18]. Liu et al. investigated localized surface plasmon resonances at mid-infrared and far-infrared wavelength regimes in BP nanoribbon and nanopatch arrays [8]. Liu et al. studied biosensor application based on plasmon-induced transparency in a BP/graphene metamaterial [15]. Li et al. fabricated field-effect transistors based on BP crystals [17]. Xia et al. investigated anisotropic conductivity for the BP layer [18]. Ti_3_C_2_T_x_ is one of the MXene 2D materials; its superior characteristics are destined to attract attention in various applications, such as sensing [19], absorption [20], and interference shielding [21]. Kim et al. discovered high-sensitivity sensing in MXene materials [19]. Morales-García et al. studied the perfect absorption phenomenon of MXene materials [20]. However, most reports on the BP layer have focused on its anisotropic properties, and most reports on MXene materials have focused on the electrochemical research of its materials. There are few studies about the excellent field enhancement properties at the interface of these two composite 2D materials. Thus, the realization of tunable light field enhancement is crucial for developing MXenes/BP-based nano-micro devices. 

In this paper, a composite MXene/BP metasurface is established for studying the transmission spectra and electric field enhancement by using the finite-difference time-domain simulation method. Firstly, the transmission spectra are investigated in the cases of only the BP layer, only the MXene layer, and both of them on the surface of substrate, respectively. We find that the Fano resonance-like spectra can be observed in the proposed composite MXene/BP metasurface. Secondly, the dependence of optical spectra and the field enhancement ratio on the structure parameters and the polarization direction of incident light for the proposed composite MXene/BP metasurface are studied. We find that both structural parameters and the polarization direction of incident light play a crucial role in the modulation of transmission spectra and field enhancement. Therefore, our findings are instructive for the design of plasmonic devices. 

## 2. Structure and Discussion 

**Figure 1:**
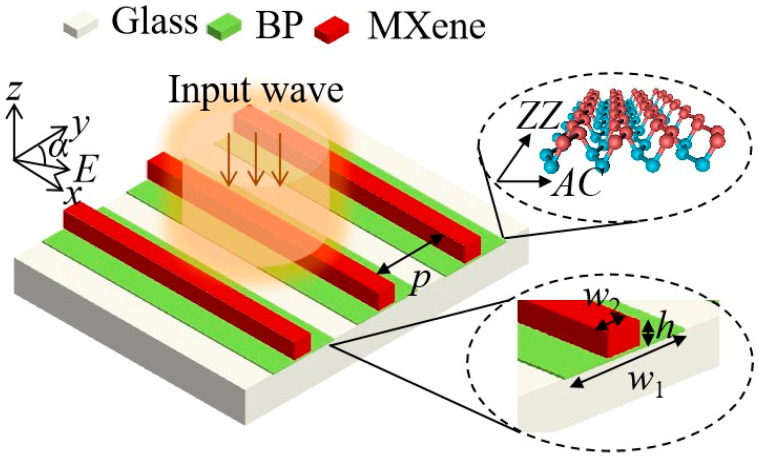
Schematic of the composite MXene/BP metasurface.

Figure 1 plots the schematic of the proposed composite MXene/BP metasurface. Here, the SiO_2_ is chosen to be the substrate for the proposed MXene/BP metasurface. The periodic BP layer is set on the substrate with the period of *p* = 500 nm. Then, the Ti_3_C_2_T_x_ MXene nanorectangular rod is placed on the BP layer as shown in Figure 1. Here, *h* is the thickness of the MXene layer, *w*_2_ is the width of the MXene layer, *w*_1_ is the width of the BP layer, and *α* is the angle between the polarization direction of the input light and the *y* axis. In the simulation, the area is divided into Yee cells with the step of Δ*x* = Δ*y* = Δ*z* = 1 nm [22]. The perfectly matched layer is chosen for the *z* direction, and periodic boundary conditions are set for the *x* and *y* directions. Here, the surface of the proposed metasurface is illuminated from the z direction by a linear polarized plane wave, as shown in Figure 1. The permittivity of SiO_2_ can be found in the reference [23]. Here, a semi-classical Drude model is introduced to describe the conductivity of the BP, expressed as [24]:

(1) $$\varepsilon_{j}=\frac{iD_{j}}{\pi (\omega +i\eta /\hbar )},\;D_{j}=\pi e^{2}\frac{n}{m_{j}}$$

where *j* = AC or ZZ direction for the anisotropic BP layer, *e* = 1.602 × 10^−19^C, *n =* 1.0 × 10^14^ cm^−2^ is the carrier density, *η* = 10 meV, and *m_AC_* and *m_ZZ_* are the effective mass for the two anisotropic directions of the BP layer, expressed as [25]:

(2) $$m_{AC}=\frac{\hbar^{2}}{\frac{2\mu^{2}}{\Delta }+\zeta },\;m_{ZZ}=\frac{\hbar^{2}}{2\nu }$$

Here, *a* = 0.223 nm, *ζ* = *ħ*^2^/(0.4*m*_0_), Δ = 2 eV, *μ* = 4*a*/*π* eVm, *ν* = *ħ*^2^/(1.4*m*_0_), and *m*_0_ = 9.10938 × 10^−31^ kg [26].

Then, a Drude–Lorentz model can be introduced for describing the permittivity of Ti_3_C_2_T_x_, expressed as [27]:

(3) $$\varepsilon_{D\mathrm{rude}}=\varepsilon_{1}-{\left(\frac{\omega_{p}}{\omega }\right)}^{2}+i(\varepsilon_{2}-\frac{\omega_{p}{}^{2}\gamma }{\omega^{3}+\omega \gamma^{2}})$$

(4) $$\varepsilon_{Lorentz}=\varepsilon_{3}\left[1+\frac{\omega_{p}{}^{2}(\omega_{0}{}^{2}-\omega^{2})+i\omega \omega_{0}{}^{2}\gamma }{{\left(\omega_{0}{}^{2}-\omega^{2}\right)}^{2}+\omega^{2}\gamma^{2}}\right]$$

where *γ* = 8.65 × 10^15^ rad/s, *ω*_p_ = 4.21 × 10^15^ rad/s, *ω*_0_ = 2.30 × 10^15^ rad/s, *ε*_1_ = 6.0, *ε*_2_ = 3.0, and *ε*_3_ = 0.2.

**Figure 2:**
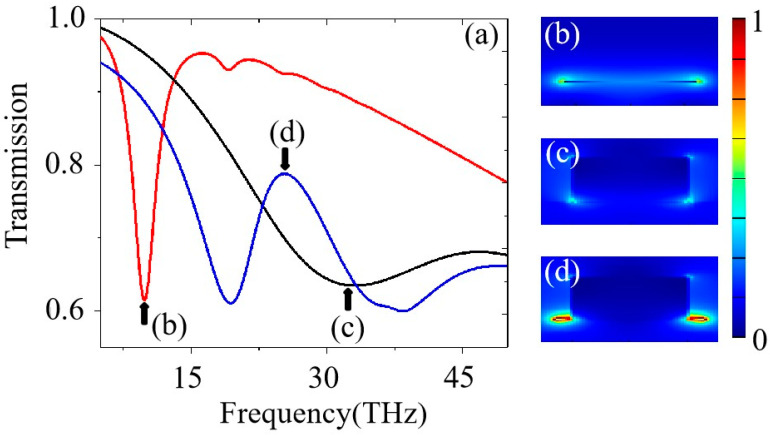
(**a**) The red line is the spectra when there is only a BP layer on the surface of SiO_2;_ the black line is the spectra when there is only a MXene nano-rod on the surface of SiO_2;_ the blue line is the spectra of the proposed composite MXene/BP metasurface. (**b**) The electric field at the frequency of 9.75 THz. (**c**) The electric field at the frequency of 32.54 THz. (**d**) The electric field at the frequency of 25.34 THz.

Here, the transmission spectra for the proposed composite MXene/BP metasurface are discussed when *h* = 150 nm, *w*_1_ = 250 nm, and *w*_2_ = 200 nm, as shown in Figure 2a. We can see that an obvious transmission dip appears at the frequency of 9.75 THz, marked in a red line, when there is only the BP layer on the surface of SiO_2_. Then, the electric field *E_B_* is studied at the frequency of 9.75 THz when there is only the BP layer on the surface of SiO_2_, as shown in Figure 2b. From Figure 2b, we can clearly see that the SPs on the surface of the BP layer are strongly excited. Therefore, the transmission dip at the frequency of 9.75 THz is caused by the strongly excited SPs on the surface of the BP layer. The black solid line shows the transmission spectra when there is only an MXene nano-rod on the surface of the SiO_2_ substrate. A transmission dip with a small quality factor occurs at the frequency of 32.54 THz, as depicted in Figure 2a. In order to further understand the physical mechanism of its generation, the electric field *E_M_*, at the frequency of 32.54 THz, is plotted in the Figure 2c. As shown in Figure 2c, strong SPs are excited on the four edges of the MXene material, and the SPs on the two edges close to the substrate are significantly stronger than those on the two edges far from the substrate. Thus, the transmission dip with the smaller quality factors is caused by the strong radiating SPs on the four edges of the MXene material, as shown in Figure 2a. At last, the blue line describes the transmission spectrum for the proposed composite MXene/BP metasurface. An obvious Fano resonance-like spectrum, caused by coupling between the mentioned two SPs modes, can be seen for the composite MXene/BP metasurface, as shown in Figure 2a. The electric field *E_M-B_*, at the frequency of 25.34 THz, for the Fano resonance-like peak is also studied in Figure 2d. Compared with the electric field in Figure 2b,c, we can see that the electric field *E_M-B_* shows strong enhancement at the interface of the MXene material and the BP layer. The strong enhancement for the electric field *E_M-B_* is caused by the strong coupling between the SPs on the BP layer and SPs on the two edges close to the substrate. Thus, the proposed composite MXene/BP metasurface can realize the Fano resonance-like spectra and obvious electric field enhancement. The findings about the Fano resonance-like spectra and electric field enhancement may be instructive for the design of plasmonic devices.

**Figure 3:**
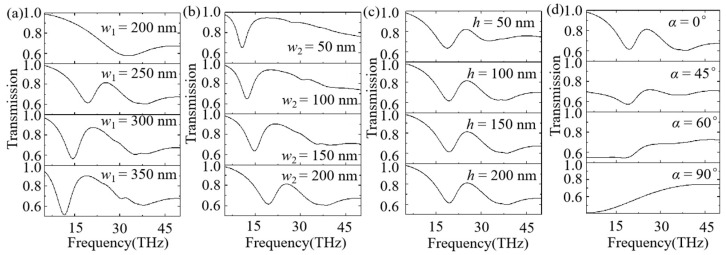
(**a**) Transmission spectra as a function of *w*_1_ with *w*_2_ = 200 nm, *h* = 150 nm, and α = 0°. (**b**) Transmission spectra as a function of *w*_2_ with *w*_1_ = 250 nm, *h* = 150 nm, and α = 0°. (**c**) Transmission spectra as a function of *h* with *w*_1_ = 250 nm, *w*_2_ = 200 nm, and α = 0°. (**d**) Transmission spectra as a function of the polarization direction of incident light α with *w*_1_ = 250 nm, *w*_2_ = 200 nm, and *h* = 150 nm.

In order to achieve modulation of transmission spectra for the proposed composite MXene/BP metasurface, the optical responses, as functions of the width *w*_1_ for the BP layer, the width *w*_2_ for the MXene layer, the thickness *h* for the MXene layer, and the polarization direction of incident light α, are studied in Figure 3. The quality factor of the Fano resonance-like spectra decreases as *w*_1_ increases from 250 nm to 350 nm, as shown in Figure 3a, which is caused by the decreasing coupling between the SPs on the surface of the BP layer and the MXene layer. Interestingly, the coupling effect disappears when *w*_1_ = *w*_2_ = 200 nm, as depicted in Figure 3a. At this time, SPs cannot be excited at the interface of the BP layer in the case of *w*_1_ = *w*_2_, so the Fano resonance-like spectra disappear. Figure 3b shows the transmission spectra as a function of the width of MXene *w*_2_. We can clearly see that the transmission spectra show a red shift as *w*_2_ increases. In addition, the results also indicate that the coupling effect increases as the width *w*_2_ decreases from 200 nm to 50 nm, and the small value of *w*_2_ corresponds to the large quality factor for transmission spectra. Moreover, the transmission for the proposed composite MXene/BP metasurface as a function of thickness *h* is investigated in Figure 3c. We can observe that the quality factor of the spectral line shows a slight increasing trend as *h* decreases from 200 nm to 50 nm. At last, the transmission spectra, as a function of the polarization direction of the incident light α with *w*_1_ = 250 nm, *w*_2_ = 200 nm, and *h* = 150 nm, are studied in Figure 3d. We can clearly see that the Fano resonance-like spectra gradually disappear as the polarization direction of incident light α increases from 0° to 90°, which is caused by the disappearing SPs on the surface of the BP and the MXene layers due to the selectivity of structure to polarized light.

**Figure 4:**
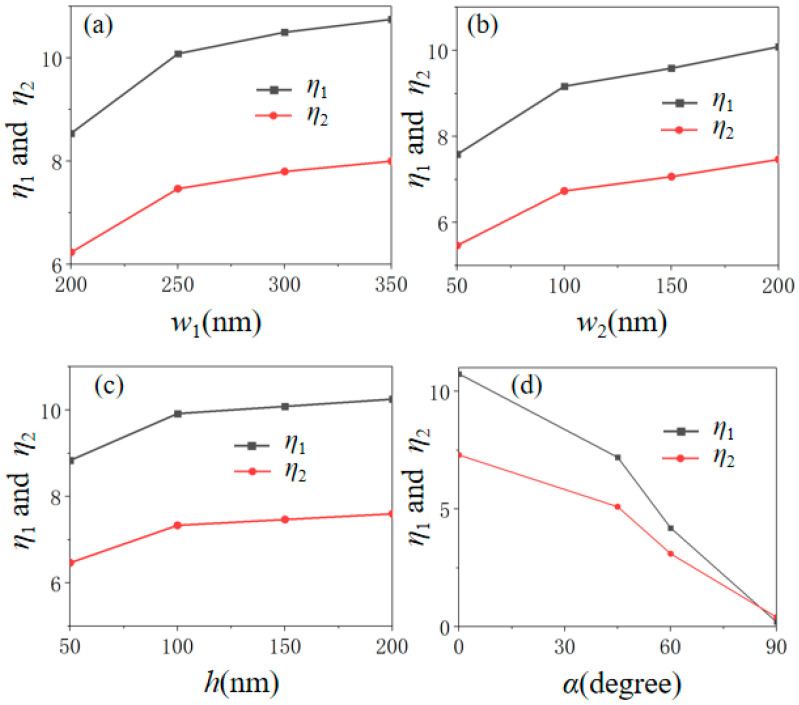
The enhancement factors *η*_1_ and *η*_2_ versus (**a**) *w*_1_, (**b**) *w*_2_, (**c**) *h*, and (**d**) *α*.

Previously, the influence of structural parameters and the polarization direction of incident light on the transmission spectra are discussed in Figure 3. Here, the influence of structural parameters and the polarization direction of incident light on the electric field enhancement characteristic are investigated, as shown in Figure 4. In order to more clearly describe the strength of the field enhancement, the enhancement factors are defined as *η*_1_ *= E_M-B_/E_B_* and *η*_2_ *= E_M-B_/E_M_* for our discussion. Here, *E_B_*, *E_M_*, and *E_M-B_* represent the value of the electric field at the wavelength of (b) point with only the BP layer, (c) point with only the MXene layer, and (d) point with both the BP and MXene layer on the surface of SiO_2_ substrate as shown in Figure 2a, respectively. From Figure 4, we can find that both the enhancement factors *η_1_* and *η_2_* increase (decrease) as *w*_1_, *w*_2_, and *h* increase (as α increases from 0° to 90°). Interestingly, the maximum enhancement factor can reach up to 10.75. Compared with other related structures [28,29,30,31], the field enhancement factor for our proposed MXene/BP-based matesurface also cannot be ignored, and the data is compared in Table 1. Therefore, the remarkable field-enhancing property has important guiding significance for enhancing the interaction between the light and matters.

## 3. Conclusions

In conclusion, a composite MXene/BP metasurface is developed for investigating transmission spectra and electric field enhancement by using the finite-difference time-domain numerical simulation method. The results show that the SPs can be defectively excited in the case of the BP layer, the MXene layer, and the composite MXene/BP layer, but the Fano resonance-like spectra characteristics can be only achieved for the proposed composite MXene/BP-based metasurface, which is caused by the coupling between the SPs on the surface of the BP layer and SPs on the surface of the MXene layer. In addition, the optical responses and electric field enhancement, as functions of the width *w*_1_, *w*_2_, *h*, and *α,* are also studied in the work. The results show that the quality factor of the Fano resonance-like spectra decreases as *w*_1_ increases from 250 nm to 350 nm, caused by the decrease of coupling between the SPs on the surface of the BP layer and the MXene layer. Interestingly, the coupling effect disappears when *w*_1_ = *w*_2_ = 200 nm. Moreover, the transmission spectra show a blue shift as the width *w*_2_ decreases from 200 nm to 50 nm. Then, we find that Fano resonance-like spectra gradually disappear as the polarization direction of incident light α increases from 0° to 90°, which is caused by the disappearing SPs on the surface of the BP and MXene layers. At last, the obvious plasmonic enhancement can be found in the microwave band caused by an interaction between the SPs on the BP layer and the MXene layer. The maximum enhancement factor can reach up to 10.75 for the proposed composite MXene/BP metasurface. The results may provide important theoretical insights for designing and realizing the high-performance plasmonic nano-micro devices.

## Figures and Tables

**Table 1:** The comparison for field enhancement between our paper and other related papers.

	Our Paper	[28]	[29]	[30]	[31]
Structure	MXene/BP	Graphene Nanoribbons	Graphene Nanoribbons	Graphene Gap	Single Metal Ring
Field Enhancement	10.75	9.2	3	10	9.5

## Data Availability

The data presented in this study are available on request from the corresponding author.
